# Evidence of increased toxic *Alexandrium tamarense* dinoflagellate blooms in the eastern Bering Sea in the summers of 2004 and 2005

**DOI:** 10.1371/journal.pone.0188565

**Published:** 2017-11-28

**Authors:** Masafumi Natsuike, Rui Saito, Amane Fujiwara, Kohei Matsuno, Atsushi Yamaguchi, Naonobu Shiga, Toru Hirawake, Takashi Kikuchi, Shigeto Nishino, Ichiro Imai

**Affiliations:** 1 Graduate School of Fisheries Sciences, Hokkaido University, Hakodate, Hokkaido, Japan; 2 Center for Marine Environmental Studies, Ehime University, Matsuyama, Ehime, Japan; 3 Japan Agency for Marine-Earth Science and Technology, Yokosuka, Kanagawa, Japan; CSIR-National Institute of Oceanography, INDIA

## Abstract

The eastern Bering Sea has a vast continental shelf, which contains various endangered marine mammals and large fishery resources. Recently, high numbers of toxic *A*. *tamarense* resting cysts were found in the bottom sediment surface of the eastern Bering Sea shelf, suggesting that the blooms have recently occurred. However, little is known about the presence of *A*. *tamarense* vegetative cells in the eastern Bering Sea. This study's goals were to detect the occurrence of *A*. *tamarense* vegetative cells on the eastern Bering Sea shelf and to find a relationship between environmental factors and their presence. Inter-annual field surveys were conducted to detect *A*. *tamarense* cells and environmental factors, such as nutrients, salinity, chlorophyll *a*, and water temperature, along a transect line on the eastern Bering Sea shelf during the summers of 2004, 2005, 2006, 2009, 2012, and 2013. *A*. *tamarense* vegetative cells were detected during every sampling year, and their quantities varied greatly from year to year. The maximum cell densities of *A*. *tamarense* observed during the summers of 2004 and 2005 were much higher than the Paralytic shellfish poisoning warning levels, which are greater than 100–1,000 cells L^-1^, in other subarctic areas. Lower quantities of the species occurred during the summers of 2009, 2012, and 2013. A significant positive correlation between *A*. *tamarense* quantity and water temperature and significant negative correlations between *A*. *tamarense* quantity and nutrient concentrations (of phosphate, silicate, and nitrite and nitrate) were detected in every sampling period. The surface- and bottom-water temperatures varied significantly from year to year, suggesting that water temperatures, which have been known to affect the cell growth and cyst germination of *A*. *tamarense*, might have affected the cells' quantities in the eastern Bering Sea each summer. Thus, an increase in the Bering Sea shelf's water temperature during the summer will increase the frequency and scale of toxic blooms and the toxin contamination of plankton feeders. This poses serious threats to humans and the marine ecosystem.

## Introduction

The Bering Sea is the northernmost marginal sea in the North Pacific and is located in the subarctic region with a vast continental shelf in its east. The shelf is greater than 500 km long from the east to the west and greater than 1,000 km long from the north to the south [1]. The shelf is home to various endangered marine mammals and large fishery resources, and sea ice covers most parts of the shelf during the winter. Climate regime shifts have been repeatedly reported in the area since at least the 1970's [2]. Warm water was observed in the area from the late 1990's to the middle of the 2000's [3,4], along with several biological changes, such as a low biomass of copepods, a coccolithophorid (*Emiliania huxleyi*) bloom, the mass mortality of short-tailed seabirds (*Puffinus tenuirostris*es), and a sudden and significant increase in large jellyfish (*Chrysaora melanaster*s) [4–7].

Paralytic shellfish poisoning (PSP) affects humans and marine animals; it is caused by consuming shellfish contaminated with toxic phytoplankton. The toxic dinoflagellate *A*. *tamarense* (Lebour) Balech is a well-known cause of PSP [8]. *A*. *tamarense* and PSP have frequently been reported from the Gulf of Alaska to south of the Aleutian Islands [9]. Additionally, *A*. *tamarense* vegetative cells have recently been reported in the Chukchi Sea shelf, and that shelf is adjacent to the Bering Sea [10]. Further, high numbers of *A*. *tamarense* resting cysts were recently found in the shelf's bottom sediment [11], and an increase in PSP toxin contaminations of marine mammals was recently reported in Alaska, ranging from the subarctic to arctic regions [12]. These previous studies suggest that *A*. *tamarense* has recently occurred in the eastern Bering Sea. However, the occurrence of *A*. *tamarense* is hardly known in the eastern Bering Sea shelf. For this study, inter-annual summer field observations were conducted to detect *A*. *tamarense* vegetative cells in a water column of the Bering Sea shelf and to evaluate the cells' connections to environmental factors.

## Materials and methods

### Field observations

**Fig 1:**
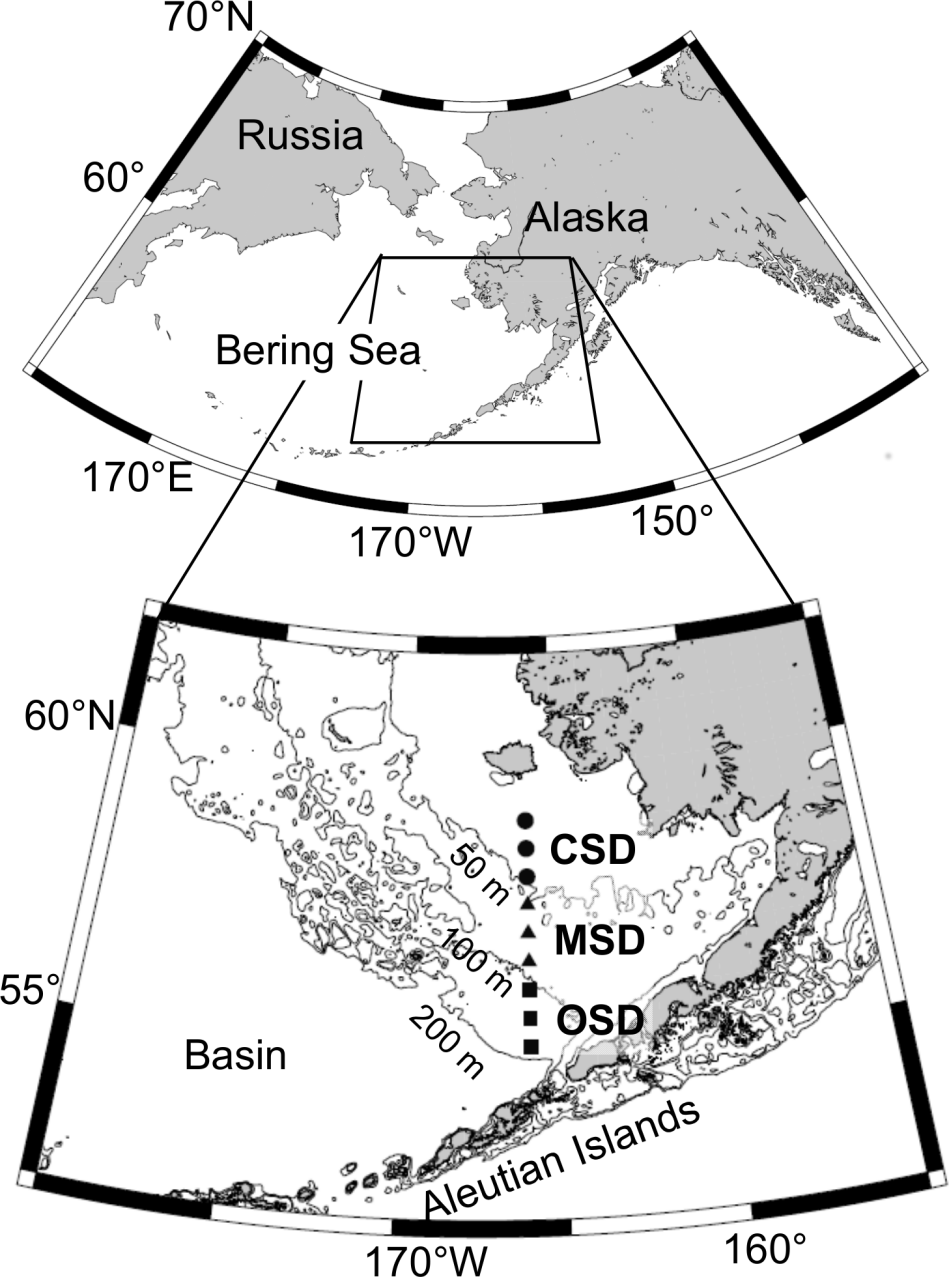
Location of the sampling stations (indicated by the black symbols) in the southeastern Bering Sea during the summers of 2004, 2005, 2006, 2009, 2012, and 2013. The sampling stations are on the 166°W transect ranging from 55° to 59°N. The study area has been divided into three domains based on hydrological features; they are the CSD, the MSD, and the OSD. Black circles indicate the stations in the CSD, triangles indicate the stations in the MSD, and squares indicate the stations in the OSD.

Field observations were performed along a transect line (166°W) from 55° to 59°N on the eastern Bering Sea shelf (Fig 1) during the summers of 2004, 2005, 2006, 2009, 2012, and 2013. The sampling stations locate in the exclusive economic zone of the USA, and the field observations were permitted by USA government and National Oceanic and Atmospheric Administration. The shelf's water column structure has been divided into three major domains according to hydrological features [1, 13]. The coastal shelf domain (CSD) is near the coast, where the water depth is less than 50 m; the water column in the CSD is not very stratified due to tidal- and wind-driven mixing. In the middle shelf domain (MSD), the water depth ranges from 50 to 100 m, and the water column is characterized by strong thermal stratification, which separates a wind-mixed surface layer and a tidal-mixed bottom layer. In the outer shelf domain (OSD), the water ranges from 100 to 200 m in depth; the surface water originates from the continental shelf, and the bottom water originates from the oceanic basin. The two waters mix to a small degree in the OSD. The 166°W transect line, ranging from 55°N to 59°N, crosses over the CSD, the MSD, and the OSD (Fig 1). It is likely *A*. *tamarense* is unable to survive in the eastern Bering Sea water column during the winter due to a lack of light, which is caused by increased sea-ice coverage and fewer daylight hours. In the coastal area to the south of the Aleutian Islands, *A*. *tamarense* occurs from late spring to summer [9]. Therefore, it was hypothesized that the dinoflagellate species mainly occurs on the eastern Bering Sea shelf during the summer.

Field observations were carried out along the 166°W transect line using the T/S *Oshoro-Maru;* the boat belongs to Hokkaido University's Faculty of Fisheries. Sampling stations were fixed at every half or one degree in latitude. Water temperature and salinity were measured using a conductivity–temperature–depth (CTD) profiler (Seabird SBE-911 plus). Seawater samples were collected from depths of 0–50 m using a plastic bucket or a Niskin rosette water sampler equipped with the CTD profiler frame. Each year's sampling dates, depths, and locations are summarized in S1 Table. The collected seawater samples were used to enumerate *A*. *tamarense* vegetative cells and measure macronutrients and chlorophyll *a* concentrations.

### Sample analysis

Immediately after collecting the samples, one liter of each seawater sample was fixed with formaldehyde or glutaraldehyde with its final concentration ranging from 0.37–1%. Fixed samples were concentrated at approximately 50–100 folds using the settling method [14]. Fluorescent dye (Calcofluor white M2R) was added to the concentrated seawater samples to stain the thecal plates of the *A*. *tamarense* vegetative cells [15]. 1–2 mL subsamples were mounted on glass slides and observed with an inverted epifluorescence microscope (Eclipse TE200) under a UV light excitation of 365 nm. This was done to identify the species morphologically and to enumerate the *A*. *tamarense* vegetative cells. The typical thecal plates of the *A*. *tamarense* vegetative cells found in each seawater sample are indicated in S1 Fig. Other seawater samples were filtered through a glass fiber filter (GF/F), and nutrient concentrations (phosphate, silicate, and nitrite and nitrate) were measured using a continuous flow analyzer (Auto Analyzer II or QuAAtro 2-HR). Chlorophyll *a* was extracted from the residue on the glass fiber filters using an organic solvent (acetone or dimethylformamide), and the concentrations were measured using a fluorometer.

To assess the coverage of sea ice during winters, sea-ice concentration data calculated by satellite observation with an SSM/I (Special Sensor Microwave/Imager) were obtained from the National Snow and Ice Data Center at the University of Colorado in the USA. The data were derived using the NASA Team algorithm [16]. Although sea-ice maps were projected onto polar stereographic coordinates with a 25 km spatial resolution, they were interpolated using the nearest-neighbor method and converted to a 9 km resolution. Then, the maximum sea-ice extent for each year was determined by defining an "ice-covered pixel" as a point where sea-ice exceeded a concentration of 10%.

### Statistical analysis

Simple regression analyses were done to assess the relationships between the quantity of *A*. *tamarense* vegetative cells and environmental factors. The analyses considered the *A*. *tamarense* cell densities and environmental factors (phosphate, salinity, chlorophyll *a*, nitrite and nitrate, water temperature, and silicate concentrations) in the seawater samples. The effects climate regime shifts and water column structures had on the *A*. *tamarense* cell densities and some of the environmental factors were evaluated using a one-way analysis of variance (ANOVA) of surface- and bottom-water temperatures and surface and bottom nutrient concentrations. The factors had significant correlations to *A*. *tamarense* cell density. A two-way ANOVA was performed using climate (warm-water periods during 2004 and 2005 and cold-water periods during 2009, 2012, and 2013) and location (the OSD and the MSD) as independent variables.

**Fig 2:**
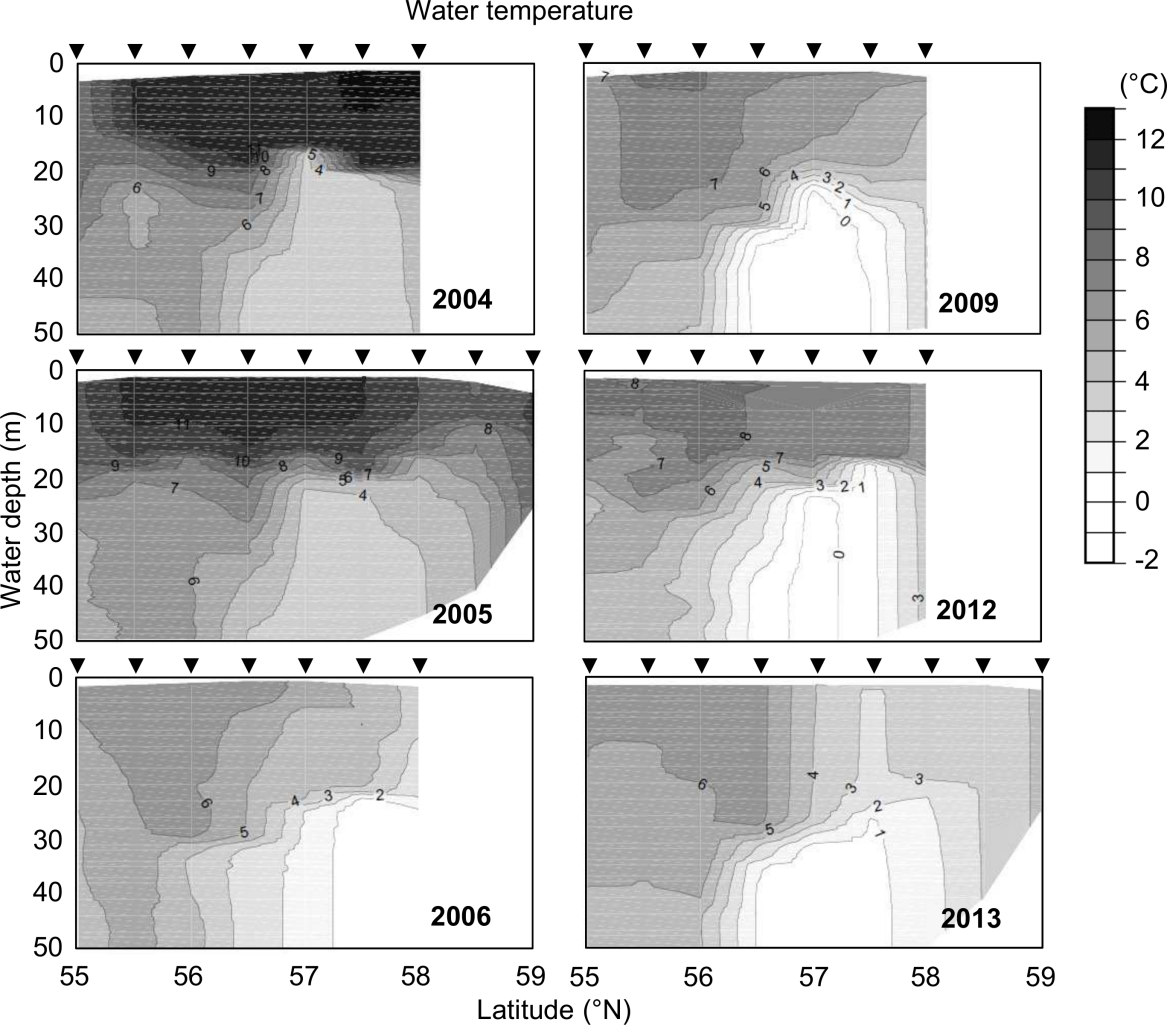
Inter-annual changes in water temperature on the 166°W transect line from 55° to 59°N and 0 to 50 m deep during the summers of 2004, 2005, 2006, 2009, 2012, and 2013.

Water temperature and nutrient concentration data were divided into surface layers (0–15 m in depth) and bottom layers (30–50 m) because their distributions were significantly different, as shown by the one-way ANOVA (*p* < 0.05). The data collected from the stations at 58.5° and 59°N (located in the CSD) during 2006 were not used for the two-way ANOVA because the sample sizes were not sufficiently large. Based on the vertical cross section of water temperature in Fig 2, the station at 58°N was on the border between the MSD and the CSD; that station's data was included in the MSD in ANOVA.

## Results

Based on inter-annual changes in water temperature along the transect line during the sampling periods (Fig 2), a climate regime shift occurred. There was a high water-temperature regime during the summers of 2004 and 2005 (a warm-water period); it changed to a low water-temperature regime during 2009, 2012, and 2013 (a cold-water period) and an intermediate water-temperature regime (between the warm- and cold-water periods) in 2006. The temperature of the surface water (a depth of 0–15 m) reached over 10°C in almost all areas during the warm-water period, but was below 8°C during the cold-water period. The temperature of the bottom water (a depth of 25 m to the bottom of the sea) in the MSD was over 3°C during the warm-water period, but was below 1°C during the cold-water period (Fig 2).

**Fig 3:**
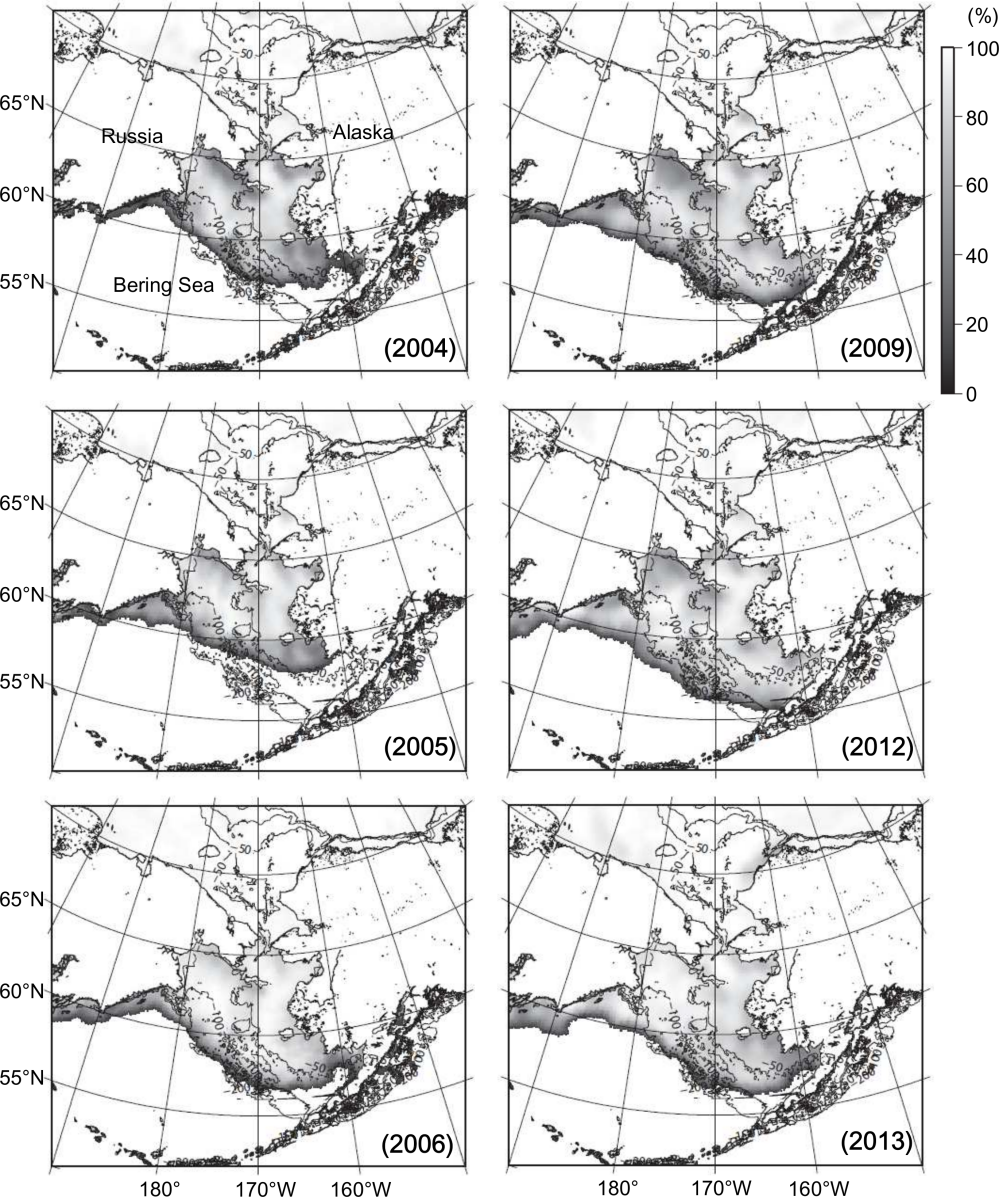
Inter-annual changes to maximum sea-ice concentrations (%) in the Bering Sea during the winters of 2004, 2005, 2006, 2009, 2012, and 2013. Sea-ice concentration data were obtained from the National Snow and Ice Data Center (see Materials and Methods). Note that from the south Bering Sea to the sea-ice covered areas there was no sea-ice cover at any point.

The maximum extents of the areas covered by sea ice during the winters of the warm- and intermediate-water periods (2004, 2005, and 2006) ranged from 8.58 × 10^5^–1.07 × 10^6^ km^2^. They were significantly smaller than those during the cold-water period (2009, 2012, and 2013), which ranged from 1.12 × 10^6^–1.27 × 10^6^ km^2^. During the warm-water period, sea ice only covered the CSD, but it reached the MSD and the OSD during the cold-water period (Fig 3).

**Fig 4:**
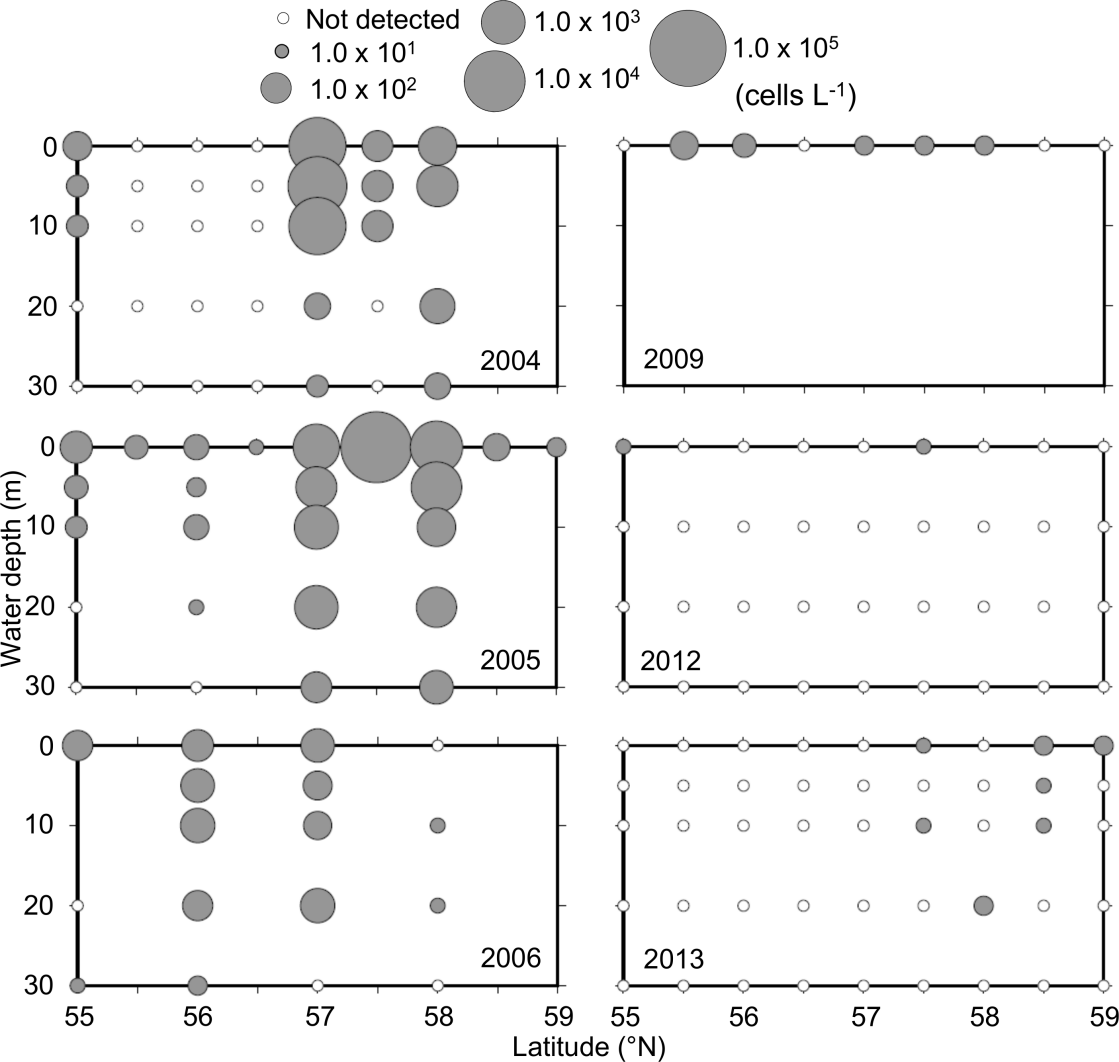
Inter-annual changes in toxic *A*. *tamarense* cell densities on the 166°W transect line from 55° to 59°N during the summers of 2004, 2005, 2006, 2009, 2012, and 2013. Note that the cell densities are shown on a common logarithmic scale. The water depths at which the seawater samples were collected are presented in S1 Table. The detection limit was 10 cells L^-1^.

*A*. *tamarense* was detected in every sampling year, but its quantity varied greatly from year to year (Fig 4). *A*. *tamarense* cells were in abundance during the warm-water period, with maximum cell densities reaching 9,450 cells L^-1^ in 2004 and 60,900 cells L^-1^ in 2005. The densities in the cold-water period (2009, 2012, and 2013) were between 10 and 80 cells L^-1^. *A*. *tamarense* occurrences were significantly higher in the MSD (at the stations at 57° and 57.5°N) and on the border between the MSD and the CSD (at the station at 58°N) during the warm-water period, as was indicated by the two-way ANOVA (*p* < 0.001) and can be seen in Fig 4.

**Fig 5:**
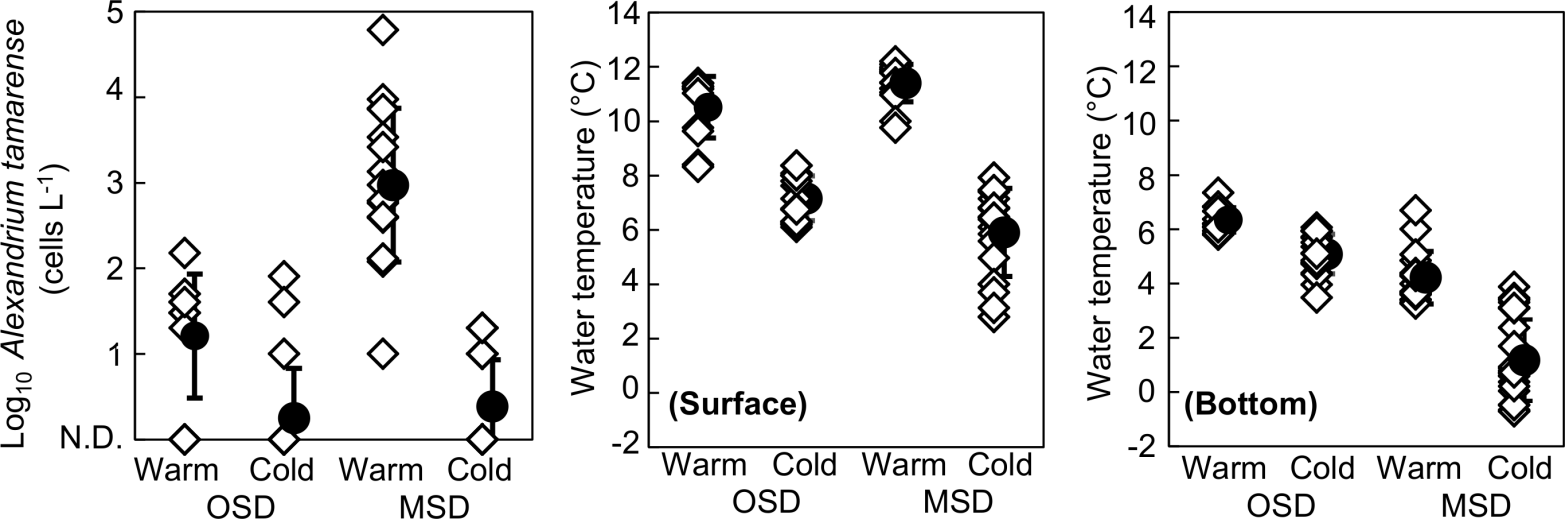
Variations in *A*. *tamarense* cell density and surface- and bottom-layer water temperatures in the OSD and the MSD during the warm and cold periods. The open symbols indicate measured values, and the error bars show the standard deviation of the fitted mean. The warm-water period was during 2004 and 2005, and the cold-water period was during 2009, 2012, and 2013. The OSD included the stations from 55° to 56°N, and the MSD included the stations from 56.5° to 58°N. The surface layer was 0–15 m deep, and the bottom layer was 30–50 m deep. The single asterisk (*) indicates the significance at 0.01 < *p* ≦ 0.05, and the double asterisks (**) indicate *p* ≦ 0.01. The sample number was 145.

A significantly positive correlation was observed between *A*. *tamarense* cell density and water temperature (*r* = 0.478, *p* < 0.001), shown in Table 1. During the warm-water period, surface-layer water temperatures (8.9°–12.2°C) were significantly higher in all sampling areas, including where *A*. *tamarense* bloomed, as was shown by the two-way ANOVA (*p* < 0.001) and can be seen in Fig 5. Bottom-layer water temperatures during the warm-water period (3.2–6.7°C) were also significantly higher than those during the cold-water period (-0.72–3.9°C), as was shown by the two-way ANOVA (*p* < 0.001) and can be seen in Fig 5. However, this increase was observed only in the MSD.

**Table 1:** Pearson's correlation coefficients (*r*) between the common logarithms of *A*. *tamarense* cell density and environmental factors (phosphate, salinity, chlorophyll *a*, nitrite and nitrate, water temperature, and dissolved silicate). The single asterisk (*) indicates the significance at 0.01 < p ≦ 0.05, and the double asterisks (**) indicate p ≦ 0.01.

Environmental Factors	*r*
Salinity	0.057
Temperature	0.478**
Chlorophyll *a*	0.042
Nitrate and nitrite	-0.228**
Phosphate	-0.288**
Silicate	-0.287**

**Fig 6:**
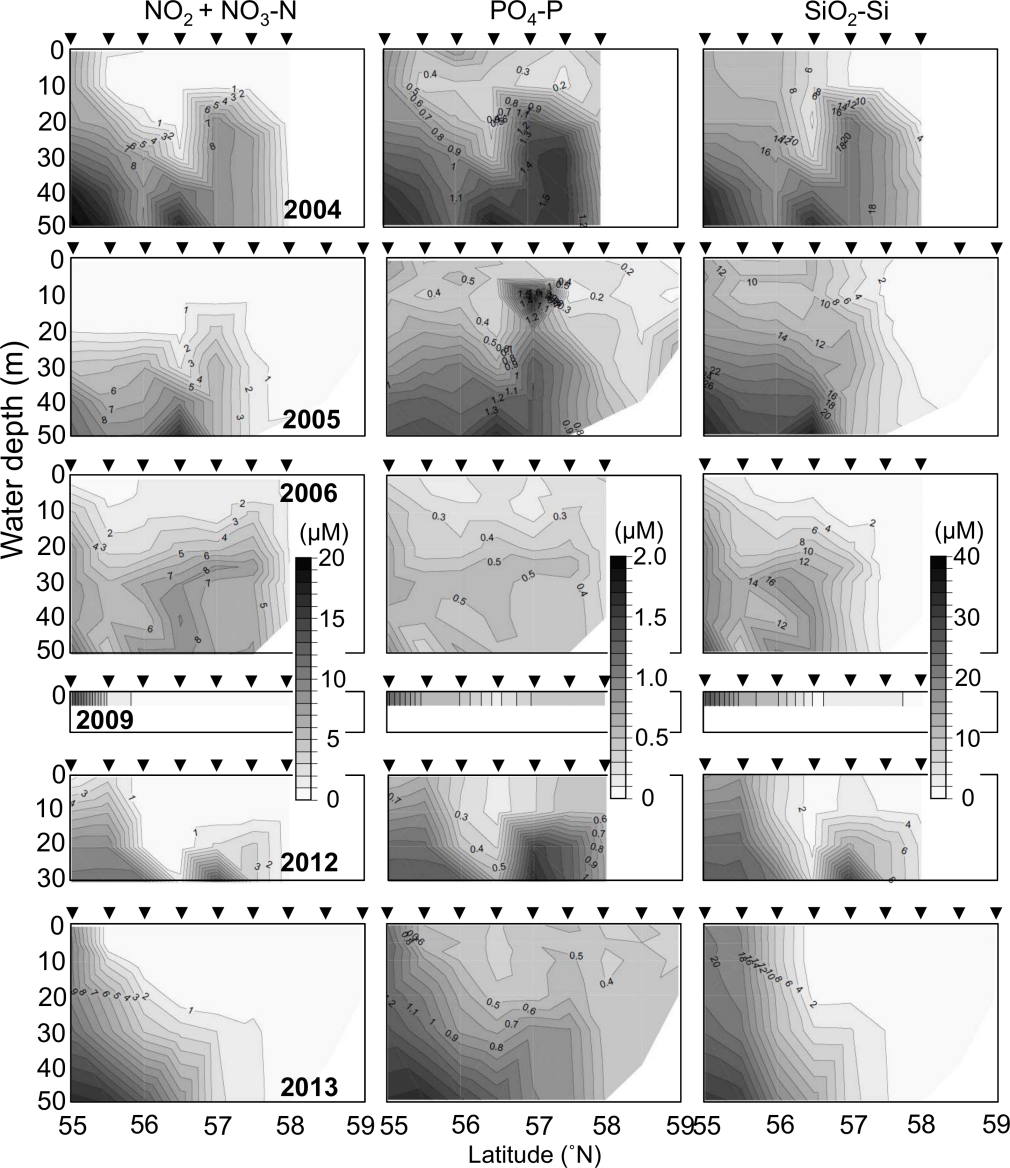
Inter-annual changes in nutrients (phosphate, silicate, and nitrite and nitrate) 0–50 m deep on the 166°W transect line from 55° to 59°N during the summers of 2004, 2005, 2006, 2009, 2012, and 2013.

Negative correlations were observed between *A*. *tamarense* cell density and phosphate, silicate, and nitrite and nitrate nutrient concentrations (-0.288, -0.287, and *r* = -0.228), respectively (*p* < 0.01), as shown in Table 1. Nutrient concentrations in the surface layer of the water were much lower than those in the bottom layer during all sampling periods, as was shown by the one-way ANOVA (*p* < 0.001) and can be seen in Fig 6. Further, nutrient concentrations in the OSD were higher than those in the MSD and the CSD in the surface and bottom layers, as was indicated by the one-way ANOVA (*p* < 0.01). However, no significant nutrient concentration differences were observed between the warm- and cold-water periods. No significant relationship was observed between salinity and chlorophyll *a*, as shown in Table 1 and S2 and S3 Figs.

## Discussion

This study revealed *A*. *tamarense* vegetative cells in the eastern Bering Sea shelf during the sampling period's summers, and the cells were relatively high in quantity during the summers of 2004 and 2005. In western Canada and northern Japan, toxin contaminations of shellfish, such as seashore mussels and seabed scallops, typically exceed regulatory levels when *A*. *tamarense* appears at densities over 100–1,000 cells L^-1^ [17, 18]. The maximum *A*. *tamarense* cell densities observed in the eastern Bering Sea during the warm periods (2004 and 2005) were approximately 10 to 60 times higher than the regulatory level. In addition, it has been reported that *A*. *tamarense* strains isolated from the eastern Bering Sea contain PSP toxins comparable to strains isolated from other PSP-occurring areas [19]. On the coast of Alaska, including the eastern Bering Sea shelf, some shellfish, such as king crabs and clams caught by fisheries and consumed by local residents in Alaska, are sometimes contaminated with PSP toxins [9]. Therefore, high toxin contaminations of shellfish were suspected in the eastern Bering Sea shelf during the warm water period.

This study confirmed the suspicion of significant inter-annual differences between the summer water temperatures of the eastern Bering Sea shelf's warm-water period (2004 and 2005) and cold-water period (2009, 2012, and 2013), as shown in Figs 2 and 5. This study also revealed spatial distributions of *A*. *tamarense* on the Bering Sea shelf during the summers of the warm-water period and the cold-water period. Although *A*. *tamarense* was present during the sampling years, its densities during the warm-water period were much higher than during the cold-water period (Figs 2 and 4). These results suggest that an increase in water temperature during the summer may promote the occurrence of this toxic species on the eastern Bering Sea shelf. This difference was also observed in sea-ice extents during winters (Fig 3). Thus, the climate regime shift from the warm-water period to the cold-water period likely occurred on the shelf during the sampling period. This climate regime shift was recognized by other studies in the same area in same period [4, 20]. Therefore, the presence of *A*. *tamarense* may be linked to the climate regime shift. However, the inter-annual surveys were conducted only once a year, and thus their seasonal occurrences are unclear. Additional seasonal detections of *A*. *tamarense* are needed to understand its dynamics and relation to the climate regime shift in the eastern Bering Sea.

This study revealed a stronger positive correlation between *A*. *tamarense* cell density and water temperature than between *A*. *tamarense* cell density and other environmental factors (salinity, chlorophyll *a*, and macronutrients). These results suggest that a high summer temperature promotes the presence of *A*. *tamarense* in the eastern Bering Sea shelf's water columns. Moreover, the toxin's bloom magnitudes during the warm-water period were higher in the MSD and the CSD than in the OSD (Fig 4). The water temperatures of the MSD and the CSD during the warm-water period were considered suitable for the growth of *A*. *tamarense*. At this location and period, the surface and bottom water temperatures were significantly higher than those of the other locations and periods. According to laboratory culture experiments from other studies [19,21,22], *A*. *tamarense* can grow at temperatures ranging from 5° to 25°C, but the optimum temperature range for its growth is 10°–22°C. The surface-water temperature ranges during the warm period were much closer to the optimum temperature than the surface-water temperature ranges during the cold period. Hence, the higher water temperatures of the surface layer, caused by the climate regime shift, encouraged the growth of *A*. *tamarense*. It has been reported that the germination rates of *A*. *tamarense* resting cysts increase with a water temperature within the range of 1°–15°C [19,23]. Moreover, it has been reported that there are more *A*. *tamarense* resting cysts in the surface sediments of the MSD than in any other areas of the eastern Bering Sea shelf [11]. Therefore, the cysts in the MSD likely germinated at high rates during the warm-water period. This likely resulted in high initial vegetative-cell densities; the cells grew in the water column and later formed toxin blooms. Thus, the higher water temperatures of the surface and bottom layers of the MSD likely led to the growth of *A*. *tamarense* in the water columns and the high germination rates of resting cysts in the bottom sediments. Together these may have made a significant contribution to the formation of the massive *A*. *tamarense* blooms in the eastern Bering Sea shelf during the summers in question. However, *A*. *tamarense* can also germinate at 1°C and grow at 5°C [19], and thus this species likely maintained its population during the cold-water period.

Significant negative correlations were observed between *A*. *tamarense* cell density and phosphate, silicate, and nitrite and nitrate nutrient concentrations, as shown in Table 1. This suggests that nutrient environments can affect *A*. *tamarense* blooms. The nutrient concentrations in the surface layer of the water were much lower than those in the bottom layer during all sampling periods (Fig 6). Further, the nutrient concentrations in the OSD were higher than those in the MSD and the CSD in the surface and bottom layers. However, no significant differences were observed between the nutrient concentrations of the warm- and cold-water periods. Therefore, the nutrient gradients observed between the surface layer and the bottom layer and the OSD and the MSD were no different than those of the warm- and cold-water periods, but the gradients were near the original water column structure in the eastern Bering Sea during the summers in question. Thus, it is unlikely that nutrient conditions were significant factors in the shelf's *A*. *tamarense* bloom magnitude. *A*. *tamarense* is known to perform diel vertical migration from the surface layer during the day to the bottom layer during the night [24,25]. This behavior enables *A*. *tamarense* to utilize the nutrients in the deeper nutrient-rich layer. Nonetheless, diatoms, which are dominant primary producers in the eastern Bering Sea [26], are non-motile and therefore do not perform diel vertical migration. The depletion of nutrients in the surface layer around the MSD may have limited the growth of diatoms during the sampling periods, but *A*. *tamarense* likely obtained supplementary nutrients from the bottom layer through vertical migration. Thus, the shortage of nutrients and the nutrient gradients of the surface and bottom layers of the MSD had the potential to encourage the growth of *A*. *tamarense* regardless of the climate regime shift.

## Conclusion

This study detected the toxin *A*. *tamarense* on the Bering Sea shelf during summer periods and revealed that the cell densities of the species drastically increased when water temperature increased. The proliferation and cyst germination of the species was likely encouraged by the increase in the surface- and bottom-water temperatures. The maximum cell densities observed during the summers of 2004 and 2005 were much higher than the PSP warning levels, which are greater than 100–1,000 cells L^-1^, of some subarctic areas. Further studies of the seasonal distribution of the species and of PSP toxin contaminations of marine organisms are needed to assess PSP incidents in the eastern Bering Sea and PSP's relation to climate regime shifts.

## Supporting information

S1 Fig Microphotographs of the thecal plates of an *Alexandrium tamarense* vegetative cell found in a seawater sample collected during field sampling. The cell was observed with an inverted epifluorescence microscope under UV light excitation (365 nm), after staining with a fluorescent dye (see Methods). All photographs show the same single cell from various angles. The white scale bars represent 20 μm. White arrows indicate the ventral pore in (A), the apical plate in (B), the anterior sulcal plate in (C), and the posterior sulcal plate in (D). All these morphological characteristics show the features of *A*. *tamarense*.
(DOCX)

S2 Fig Seasonal changes in chlorophyll a at a depth of 0–50 m on the 166°W transect from 55 to 59°N, during the summers of 2004, 2005, 2006, 2009, 2012, and 2013. (DOCX)

S3 Fig Seasonal changes in salinity at a depth of 0–50 m on the 166°W transect from 55 to 59°N, during the summers of 2004, 2005, 2006, 2009, 2012, and 2013. (DOCX)

S1 Table Sampling stations (cruise and station numbers of T/V *Oshoro-Maru*), location, dates, depths (m), and cell densities of *Alexandrium tamarense* of the seawater samples collected during field sampling on the 166° transect line in the eastern Bering Sea, during 2004, 2005, 2006, 2009, 2012, and 2013. (DOCX)
