# Supplementary material for: Evidence of increased toxic Alexandrium tamarense dinoflagellate blooms in the eastern Bering Sea in the summers of 2004 and 2005
Source: PLoS One. 2017 Nov 28;12(11):e0188565. doi: 10.1371/journal.pone.0188565 (PMC5705126; doi:10.1371/journal.pone.0188565)
Supplement: S1 Fig — The cell was observed with an inverted epifluorescence microscope under UV light excitation (365 nm), after staining with a fluorescent dye (see Methods). All photographs show the same single cell from various angles. The white scale bars represent 20 μm. White arrows indicate the ventral pore in (A), the apical plate in (B), the anterior sulcal plate in (C), and the posterior sulcal plate in (D). All these morphological characteristics show the features of A. tamarense. (DOCX) [file pone.0188565.s001.docx]

**S1 Fig.** **Microphotographs of the thecal plates of an *Alexandrium tamarense* vegetative cell found in a seawater sample collected during field sampling.** The cell was observed with an inverted epifluorescence microscope under UV light excitation (365 nm), after staining with a fluorescent dye (see Methods). All photographs show the same single cell from various angles. The white scale bars represent 20 µm. White arrows indicate the ventral pore in (**A**), the apical plate in (**B**), the anterior sulcal plate in (**C**), and the posterior sulcal plate in (**D**). All these morphological characteristics show the features of *A. tamarense*.
