# Supplementary material for: Evidence of increased toxic Alexandrium tamarense dinoflagellate blooms in the eastern Bering Sea in the summers of 2004 and 2005
Source: PLoS One. 2017 Nov 28;12(11):e0188565. doi: 10.1371/journal.pone.0188565 (PMC5705126; doi:10.1371/journal.pone.0188565)
Supplement: S3 Fig — (DOCX) [file pone.0188565.s003.docx]

**S3 Fig.** **Seasonal changes in salinity at a depth of 0 – 50 m on the 166°W transect from 55 to 59°N, during the summers of 2004, 2005, 2006, 2009, 2012, and 2013.**
