# Supplementary material for: Evidence of increased toxic Alexandrium tamarense dinoflagellate blooms in the eastern Bering Sea in the summers of 2004 and 2005
Source: PLoS One. 2017 Nov 28;12(11):e0188565. doi: 10.1371/journal.pone.0188565 (PMC5705126; doi:10.1371/journal.pone.0188565)
Supplement: S1 Table — (DOCX) [file pone.0188565.s004.docx]

**S1 Table. Sampling stations (cruise and station numbers of T/V *Oshoro-Maru*), location, dates, depths (m), and cell densities of *Alexandrium tamarense* of the seawater samples collected during field sampling on the 166° transect line in the eastern Bering Sea, during 2004, 2005, 2006, 2009, 2012, and 2013.**

| Cruise No. | Station No. | Latitude (°N) | Longitude (°W) | Date (M/D/Y) | Water depth (m) | *Alexandrium tamarense* (cells L^-1^) |
| --- | --- | --- | --- | --- | --- | --- |
| C143 | OS04129 | 55.0 | 166.0 | 7/28/2004 | 0 | 90 |
|  |  |  |  |  | 5 | 30 |
|  |  |  |  |  | 10 | 30 |
|  |  |  |  |  | 20 | Not detected |
|  |  |  |  |  | 30 | Not detected |
|  | OS04131 | 56.0 | 166.0 | 7/29/2004 | 0 | Not detected |
|  |  |  |  |  | 5 | Not detected |
|  |  |  |  |  | 10 | Not detected |
|  |  |  |  |  | 20 | Not detected |
|  |  |  |  |  | 30 | Not detected |
|  | OS04133 | 57.0 | 166.0 | 7/29/2004 | 0 | 7350 |
|  |  |  |  |  | 5 | 9450 |
|  |  |  |  |  | 10 | 7200 |
|  |  |  |  |  | 20 | 60 |
|  |  |  |  |  | 30 | 30 |
|  | OS04134 | 57.5 | 166.0 | 7/29/2004 | 0 | 120 |
|  |  |  |  |  | 5 | 130 |
|  |  |  |  |  | 10 | 130 |
|  |  |  |  |  | 20 | Not detected |
|  |  |  |  |  | 30 | Not detected |
|  | OS04135 | 58.0 | 166.0 | 7/29/2004 | 0 | 390 |
|  |  |  |  |  | 5 | 610 |
|  |  |  |  |  | 20 | 230 |
|  |  |  |  |  | 30 | 60 |
| C159 | OS05075 | 55.0 | 166.0 | 7/14/2005 | 0 | 150 |
|  |  |  |  |  | 5 | 40 |
|  |  |  |  |  | 10 | 30 |
|  |  |  |  |  | 20 | Not detected |
|  |  |  |  |  | 30 | Not detected |
|  | OS05076 | 55.5 | 166.0 | 7/14/2005 | 0 | 40 |
|  | OS05077 | 56.0 | 166.0 | 7/15/2005 | 0 | 50 |
|  |  |  |  |  | 5 | 20 |
|  |  |  |  |  | 10 | 50 |
|  |  |  |  |  | 20 | 10 |
|  |  |  |  |  | 30 | Not detected |
|  | OS05078 | 56.5 | 166.0 | 7/15/2005 | 0 | 10 |
|  | OS05079 | 57.0 | 166.0 | 7/15/2005 | 0 | 1340 |
|  |  |  |  |  | 5 | 580 |
|  |  |  |  |  | 10 | 940 |
|  |  |  |  |  | 20 | 850 |
|  |  |  |  |  | 30 | 120 |
|  | OS05080 | 57.5 | 166.0 | 7/15/2005 | 0 | 60900 |
|  | OS05081 | 58.0 | 166.0 | 7/15/2005 | 0 | 3400 |
|  |  |  |  |  | 5 | 2600 |
|  |  |  |  |  | 10 | 400 |
|  |  |  |  |  | 20 | 520 |
|  |  |  |  |  | 30 | 190 |
|  | OS05082 | 58.5 | 166.0 | 7/16/2005 | 0 | 70 |
|  | OS05083 | 59.0 | 166.0 | 7/16/2005 | 5 | 20 |
| C169 | OS06131 | 55.0 | 166.0 | 6/21/2006 | 0 | 110 |
|  |  |  |  |  | 20 | Not detected |
|  |  |  |  |  | 30 | 10 |
|  | OS06133 | 56.0 | 166.0 | 6/21/2006 | 0 | 140 |
|  |  |  |  |  | 5 | 190 |
|  |  |  |  |  | 10 | 210 |
|  |  |  |  |  | 20 | 110 |
|  |  |  |  |  | 30 | 20 |
|  | OS06135 | 57.0 | 166.0 | 6/22/2006 | 0 | 180 |
|  |  |  |  |  | 5 | 90 |
|  |  |  |  |  | 10 | 80 |
|  |  |  |  |  | 20 | 210 |
|  |  |  |  |  | 30 | Not detected |
|  | OS06137 | 58.0 | 166.0 | 6/22/2006 | 0 | Not detected |
|  |  |  |  |  | 10 | 10 |
|  |  |  |  |  | 20 | 10 |
|  |  |  |  |  | 30 | Not detected |
| C202 | OS09119 | 55.0 | 166.0 | 7/8/2009 | 0 | Not detected |
|  | OS09120 | 55.5 | 166.0 | 7/8/2009 | 0 | 80 |
|  | OS09121 | 56.0 | 166.0 | 7/9/2009 | 0 | 40 |
|  | OS09127 | 56.5 | 166.0 | 7/10/2009 | 0 | Not detected |
|  | OS09128 | 57.0 | 166.0 | 7/10/2009 | 0 | 20 |
|  | OS09131 | 57.5 | 166.0 | 7/11/2009 | 0 | 20 |
|  | OS09133 | 58.0 | 166.0 | 7/11/2009 | 0 | 20 |
| C243 | OS12117 | 55.0 | 166.0 | 7/25/2012 | 0 | 10 |
|  |  |  |  |  | 10 | Not detected |
|  |  |  |  |  | 20 | Not detected |
|  |  |  |  |  | 30 | Not detected |
|  | OS12035 | 55.5 | 166.0 | 7/25/2012 | 0 | Not detected |
|  |  |  |  |  | 10 | Not detected |
|  |  |  |  |  | 20 | Not detected |
|  |  |  |  |  | 30 | Not detected |
|  | OS12119 | 56.0 | 166.0 | 7/25/2012 | 0 | Not detected |
|  |  |  |  |  | 10 | Not detected |
|  |  |  |  |  | 20 | Not detected |
|  |  |  |  |  | 30 | Not detected |
|  | OS12120 | 56.5 | 166.0 | 7/25/2012 | 0 | Not detected |
|  |  |  |  |  | 10 | Not detected |
|  |  |  |  |  | 20 | Not detected |
|  |  |  |  |  | 30 | Not detected |
|  | OS12121 | 57.0 | 166.0 | 7/26/2012 | 0 | Not detected |
|  |  |  |  |  | 10 | Not detected |
|  |  |  |  |  | 20 | Not detected |
|  |  |  |  |  | 30 | Not detected |
|  | OS12122 | 57.5 | 166.0 | 7/26/2012 | 0 | 10 |
|  |  |  |  |  | 10 | Not detected |
|  |  |  |  |  | 20 | Not detected |
|  |  |  |  |  | 30 | Not detected |
|  | OS12123 | 58.0 | 166.0 | 7/26/2012 | 0 | Not detected |
|  |  |  |  |  | 10 | Not detected |
|  |  |  |  |  | 20 | Not detected |
| C255 | OS13058 | 55.0 | 166.0 | 6/26/2013 | 0 | Not detected |
|  |  |  |  |  | 5 | Not detected |
|  |  |  |  |  | 10 | Not detected |
|  |  |  |  |  | 20 | Not detected |
|  |  |  |  |  | 30 | Not detected |
|  | OS13057 | 55.5 | 166.0 | 6/26/2013 | 0 | Not detected |
|  |  |  |  |  | 5 | Not detected |
|  |  |  |  |  | 10 | Not detected |
|  |  |  |  |  | 20 | Not detected |
|  |  |  |  |  | 30 | Not detected |
|  | OS13056 | 56.0 | 166.0 | 6/26/2013 | 0 | Not detected |
|  |  |  |  |  | 5 | Not detected |
|  |  |  |  |  | 10 | Not detected |
|  |  |  |  |  | 20 | Not detected |
|  |  |  |  |  | 30 | Not detected |
|  | OS13055 | 56.5 | 166.0 | 6/26/2013 | 0 | Not detected |
|  |  |  |  |  | 5 | Not detected |
|  |  |  |  |  | 10 | Not detected |
|  |  |  |  |  | 20 | Not detected |
|  |  |  |  |  | 30 | Not detected |
|  | OS13054 | 57.0 | 166.0 | 6/26/2013 | 0 | Not detected |
|  |  |  |  |  | 5 | Not detected |
|  |  |  |  |  | 10 | Not detected |
|  |  |  |  |  | 20 | Not detected |
|  |  |  |  |  | 30 | Not detected |
|  | OS13053 | 57.5 | 166.0 | 6/25/2013 | 0 | 10 |
|  |  |  |  |  | 5 | Not detected |
|  |  |  |  |  | 10 | 10 |
|  |  |  |  |  | 20 | Not detected |
|  |  |  |  |  | 30 | Not detected |
|  | OS13052 | 58.0 | 166.0 | 6/25/2013 | 0 | Not detected |
|  |  |  |  |  | 5 | Not detected |
|  |  |  |  |  | 10 | Not detected |
|  |  |  |  |  | 20 | 20 |
|  |  |  |  |  | 30 | Not detected |
|  | OS13051 | 58.5 | 166.0 | 6/25/2013 | 0 | 20 |
|  |  |  |  |  | 5 | 10 |
|  |  |  |  |  | 10 | 10 |
|  |  |  |  |  | 20 | Not detected |
|  |  |  |  |  | 30 | Not detected |
|  | OS13050 | 59.0 | 166.0 | 6/25/2013 | 0 | 20 |
|  |  |  |  |  | 5 | Not detected |
|  |  |  |  |  | 10 | Not detected |
|  |  |  |  |  | 20 | Not detected |
